# Depression-Like Behavioral Phenotypes by Social and Social Plus Visual Isolation in the Adult Female *Macaca fascicularis*

**DOI:** 10.1371/journal.pone.0073293

**Published:** 2013-09-04

**Authors:** Xin Li, Fan Xu, Liang Xie, Yongjia Ji, Ke Cheng, Qinmin Zhou, Tao Wang, Carol Shively, Qingyuan Wu, Wei Gong, Liang Fang, Qunlin Zhan, N. D. Melgiri, Peng Xie

**Affiliations:** 1 Department of Neurology, the First Affiliated Hospital of Chongqing Medical University, Chongqing, China; 2 Institute of Neuroscience, Chongqing Medical University, and Chongqing Key Laboratory of Neurobiology, Chongqing, China; 3 Department of Neurology, the Fifth People’s Hospital, Chongqing, China; 4 Department of Pathology, Wake Forest School of Medicine, Winston-Salem, North Carolina, United States of America; McGill University, Canada

## Abstract

Major depressive disorder (MDD) is a debilitating psychiatric mood disorder that affects millions of individuals globally. Our understanding of the biological basis of MDD is poor, and current treatments are ineffective in a significant proportion of cases. This current situation may relate to the dominant rodent animal models of depression, which possess translational limitations due to limited homologies with humans. Therefore, a more homologous primate model of depression is needed to advance investigation into the pathophysiological mechanisms underlying depression and to conduct pre-clinical therapeutic trials. Here, we report two convenient methods – social isolation and social plus visual isolation – which can be applied to construct a non-human primate model of depression in the adult female cynomolgus monkey (*Macaca fascicularis*). Both social and social plus visual isolation were shown to be effective in inducing depression-like behavior by significantly reducing socially dominant aggressive conflict behavior, communicative behavior, sexual behavior, and parental behavior. The addition of visual isolation produced more profound behavioral changes than social isolation alone by further reducing parental behavior and sexual behavior. Thus, the degree of behavioral pathology may be manipulated by the degree of isolation. These methods can be applied to construct a non-human primate model of depression in order to assess physiological, behavioral, and social phenomena in a controlled laboratory setting.

## Introduction

Major depressive disorder (MDD, major depression), which affects up to 25% of women and 12% of men worldwide, is a debilitating neuropsychiatric mood disorder of unclear etiology. [1] However, it is well-established that stressful life events increase the risk of depression. Moreover, as early life stressors predispose individuals to depression in later life, stressors may precipitate a depressive episode proximally or distally and are also associated with recurrences. [2].

One life stressor, social isolation (defined as an absence or low frequency of peer interaction), [3] has been associated with an increased risk of depression and anxiety. [4] Social isolation leads to passive responses to stress and subjective feelings of hopelessness and helplessness that increase the risk of depression and anxiety. [5] Long-term social isolation also molds eccentric social characteristics, further compounding social isolation and its deleterious effects. [6].

Therefore, social isolation has been used to construct animal models of depression. [7] In rodent models, social isolation produces negative affective changes (e.g., anhedonia, psychomotor retardation, neophobia, aggression) and neurophysiological effects similar to those observed in human mood disorders (e.g., hypothalamic–pituitary–adrenal (HPA) axis modulation, decreased brain-derived neurotrophic factor [BDNF] expression). [8]–[10] Although these rodent models have been constructive to our current understanding of depression, the greater structural and functional central nervous system (CNS) homologies between the macaque and human suggest that a macaque model of depression can further aid future neuropsychiatric investigation. Thus, social isolation has also been used to construct macaque models of depression. Harlow *et al.* first used varying degrees of social separation and isolation to induce a depression-like response in juvenile macaques. [11] However, psychosocially immature juvenile macaques may not be able to express the full range of affective and social behaviors needed to model depression, [12] and the accumulation of mild, chronic stressors in adulthood more closely approximates the etiopathological development of depression; [13] thus, adult macaques appear to be a better fit for modeling depression. Our previous work constructed a systematic ethogram of the adult female cynomolgus monkey (*Macaca fascicularis)* reared and observed in a social environment, demonstrating that these subjects were psychosocially mature. [14] Moreover, a few research groups have begun social isolation studies on adult female macaques, and these investigations have revealed depression-like characteristics (e.g., anhedonia, increased rates of submissive behaviors, and reduced hippocampal neurogenesis) in socially-isolated adult macaques. [15], [16].

Here, we sought to examine and compare the effects of two levels of social isolation on adult female macaques. Since visual contact is a primary modality of social communication in primates, [17] the behavioral effects of both social and social plus visual isolation were assessed by removing monkeys from their long term stable social groups and housing them in standard single cages (SSC, social isolation) or in single cages with no visual contact (NVC, social plus visual isolation). We hypothesized that social and social plus visual isolation would result in deficits in social interaction and a depression-like state, and that social plus visual isolation would produce more profound effects than social isolation alone.

## Methods

### Subjects

Thirty healthy adult female *M. fascicularis* subjects (aged 9–13 years) were randomly selected from a pool of 6012 monkeys. All subjects were reared in socially stable colonies with negligible rates of conflict. Each colony was housed in an indoor free enclosure measuring 8.0×3.0×3.0 m (L×W×H) with continuous daylight exposure. Every colony was composed of 2 males, 16–22 adult females, and their offspring of less than 6 months of age. To reflect wild populations, the male:female ratio was maintained at 1∶7–11.

### Ethics Statement

All animal work, in both the single-cage and free enclosure environments, was conducted according to relevant national and international guidelines. In accordance with the recommendations of the Weatherall report, “The use of non-human primates in research,” the following statement has been included to document the details of animal welfare and the steps taken to ameliorate suffering in all work involving non-human primates. This study was performed in strict accordance with the recommendations in the “Guide for the Care and Use of Laboratory Animals” of the Institute of Neuroscience at Chongqing Medical University (Approval No.: 20100031). State regulators and the Committee on Ethics of Animal Experimentation at Chongqing Medical University approved the protocols for both the single-cage and free enclosure environments prior to implementation.

Single cages were installed in specialized quarantined cage-breeding houses for the convenience of the veterinary and other staff’s inspection of individual subjects. Specifically, 36 single cages were installed per cage-breeding house with one cage-breeding house per subject group. As with control subjects, single-caged subjects were provided water *ad libitum* through a tube and fed twice daily with fresh fruit and vegetables and compound high-nutrition monkey food. Veterinarians and feeders conducted daily checks on the single-caged subjects; if needed, every subject received immediate treatment to minimize harm. For more detail on the free enclosure environment, please refer to our previous publication. [14].

For the purposes of environmental enrichment, the free enclosure environment included a shelf and ring affixed to the skylight and toy balls for playing, all of which were popular with the monkeys. In the single-cage environment, round mirrors were affixed onto the side of each individual cage, and a toy ball was also provided to each subject.

### Experimental Procedures

Immediately after weighing, a pre-isolation observation of all subjects conducted over three consecutive days was performed in the subjects’ respective free enclosures. Then, the 30 subjects were randomly divided into three groups: a socially isolated standard single cage (SSC) group (n = 5), a ‘no visual contact’ (NVC) single cage group (n = 5), and a socially housed control (control) group (n = 20). As infants under six months of age are required to be reared by their mothers according to AAALAC requirements, SSC and NVC subjects did not possess offspring and were ruled-out for pregnancy; however, eight of the twenty control subjects did possess offspring (Table 1).

**Table 1:** Group Data.

Variable	SSC	NVC	Control	*P*-value
Sample size (n)	5	5	20	–
Age (yr)*	12.20±0.74	12.20±0.66	11.65±1.90	0.15
Pre-isolationweight (kg)*	5.81±0.76	5.55±0.59	5.93±0.88	0.37
Post-isolationweight (kg)*	6.23±0.68	5.97±0.83	6.01±0.74	0.17
Offspring	0	0	8	–

* Data presented as means ± S.D.’s. For all three groups, there was no significant difference in pre- vs. post-isolation weights (*p*>0.05).

Control subjects remained in their respective free enclosures throughout the entire study. Immediately after pre-isolation observation, SSC and NVC subjects were removed from their respective free enclosures and placed in individual standard single cages (measuring 0.6×0.7×0.8 m) together in one cage-breeding house for a 14-day habituation period. Then, NVC subjects were moved into five individual standard single cages retrofitted with opaque walls (NVC cages) in a separate cage-breeding house for a 90-day social plus visual isolation period, while the SSC subjects remained in their standard single cages in the original cage-breeding house. Thus, SSC subjects, while socially isolated, remained in visual, olfactory, and auditory contact with other SSC subjects in the room, whereas NVC subjects were socially isolated and had no visual contact, but retained olfactory and auditory contact, with the other subjects in the cage-breeding house.

After the 90-day isolation period, SSC and NVC subjects were immediately weighed and returned to their respective colonies. The colony members would inspect the returning subjects, and after five days, the SSC and NVC subjects were fully re-integrated into the normal social interactions of the colony. After this five-day habituation period, three consecutive days of post-isolation observation was conducted on all subjects.

### Behavioral Paradigm, Observation, and Recording

Based on the Posture-Action-Environment principle, our lab group previously validated and classified 53 discrete behaviors of 40 young-adult female *M. fascicularis* subjects into 12 behavioral categories in order to construct a systematic ethogram for the species. [14] These behavioral items and categories have been applied in the current study. As to recording, all behavior was videotaped with one day of observation consisting of seven observational phases of 30 minutes each as follows: A2 10:00–10∶30, A3 10:30–11∶00, A4 11:00–11∶30, P2 14:30–15∶00, P3 15:00–15∶30, P4 15:30–16∶00, and P5 16:00–16∶30. Three trained observers blindly scored the durations (in seconds) of all discrete behavioral items on the videotape footage using NOLDUS Observer XT software (version 10.0, Noldus Information Technology, Leesburg, PA). [18] Data on all discrete behavioral items in 12 behavioral categories were gathered (Table 2, File S1) with an inter-observer reliability of greater than 85% for each discrete behavioral item.

**Table 2:** Descriptions of Behavioral Categories and Items.

Behavioral Categories∧	Behavioral Items and Definitions
Ingestion	Licking residue from floor (licking of food residue scattered on the floor)
Thermo-regulatory	Embracing (embracing another subject)
Rutting and estrous (sexual)	Presenting buttocks (presenting the buttocks to another subject)
	Same-sex mounting (pelvic mounting of another subject observed during same-sex encounters)
Mating (sexual)	Copulating (sexual intercourse between heterosexual subjects)
Resting	Lying on floor (lying on the floor)
	Hanging on window or door (hanging on the window or door)
Parental	Nursing infant (nursing an infant)
	Holding infant (holding an infant)
Amicable (affiliative)	Grooming (grooming another subject)
	Being groomed (grooming by another subject)
	Embracing (embracing another subject)
Conflict	Threatening (threatening another subject including staring, opening the mouth, and/or baring the teeth)
Vigilance	Watching company (keeping watch of other subjects)
	Miscellaneous calling (calling behavior not seemingly directed to another subject or to the group)
Locomotive	Walking on shelf (walking on the shelf, an elevated perch)
	Quadrupedal walking on floor (walking on the floor on all four limbs)
Communication	Lip smacking (smacking of the lips in communicating with another subject)
	Miscellaneous calling (calling behavior not seemingly directed to another subject or to the group)
Miscellaneous (self-directed)	Huddling (huddling [self-embracing] on the floor)
	Playing (solitary playing with a toy)
	Licking hair (self-licking of the subject’s hair)
	Licking tail (self-licking of the subject’s tail)
	Shaking ID card (shaking the ID card placed around the subject’s neck)

∧ More detail behavioral items and categories established by systematic ethogram of *M. fascicularis* [14].

### Data Analysis

Behavioral data were coded as duration (in seconds) for each discrete behavioral item per each 30-minute observational phase and presented as means ± S.D.’s (for raw data, please see File S1.). To rule out behaviors not induced by social (SSC) or social plus visual (NVC) isolation, all pre-isolation and post-isolation behavioral items were compared by the Mann–Whitney U (Wilcoxon rank-sum) test. Then, all pre-isolation or post-isolation behavioral items were analyzed by a 1 (pre- or post-isolation)×3 (control, SSC, and NVC) mixed analysis of variance (ANOVA) model to compare the behaviors of NVC and SSC groups with those of non-isolated controls. In order to improve accuracy, the α-level was modified according to the quantity of variables (specifically, α′ = α/15 = 0.0033). *P*-values<0.0033 were deemed significant for all analyses. All data management and statistical analysis were performed using Stata 12.0 (StataCrop LP, College Station, Texas 77845 USA).

## Results

There was no significant differences in age or weight between the SSC, NVC, and control subjects (*p*>0.05) (Table 1). Isolation had no significant effects on the weights of the SSC and NVC subjects (*p*>0.05) (Table 1).

### Behavioral Effects of Social Isolation (SSC) and Social Plus Visual Isolation (NVC)

Social isolation significantly reduced five behaviors in SSC subjects (in order of decreasing fold-change magnitude): ‘threatening’ (a conflict behavior), ‘embracing’ (an amicable behavior), ‘presenting buttocks’ (a sexual behavior), ‘lip smacking’ (a communication behavior), and ‘walking on shelf’ (a locomotive behavior) (Table 3). In contrast, social isolation significantly increased two behaviors (in order of decreasing fold-change magnitude): ‘playing’ (a self-directed behavior) and ‘being groomed’ (an amicable behavior) (Table 3).

**Table 3:** Differential Behaviors in SSC Subjects Ranked by Fold-Change Magnitude.

Behavioral Item∧	Behavioral Category∧	Pre-isolation (s)†	Post-isolation (s)†	*P*-value*
Licking residue from floor	Ingestion	3.34±30.87	0.96±7.54	0.0045
Holding infant	Parental	149.07±399.54	48.21±213.66	0.0077
Threatening	Conflict	0.48±4.16	0.19±1.17	0.0002#
Embracing (w/conspecific)	Amicable (affiliative)	257.62±431.83	123.38±324.91	0.0001#
Presenting buttocks	Rutting and estrous (sexual)	3.39±19.09	2.05±16.42	0.0000#
Lip smacking	Communication	4.29±48.95	2.77±34.24	0.0010#
Miscellaneous calling	Communication, vigilance	0.25±3.34	0.19±2.76	0.0212
Walking on shelf	Locomotive	5.58±12.80	4.39±11.20	0.0010#
Lying on floor	Resting	15.03±83.73	51.75±170.94	0.0042
Licking tail	Self-directed	0.19±1.52	0.53±3.73	0.0392
Quadrupedal walking on floor	Locomotive	67.57±64.28	82.33±73.85	0.0112
Playing (solitary)	Self-directed	2.47±19.63	2.77±20.30	0.0001#
Watching company	Vigilance	1239.08±423.36	1280.70±416.11	0.0263
Grooming	Amicable (affiliative)	93.62±198.16	96.66±213.41	0.0359
Being groomed	Amicable (affiliative)	100.65±195.53	100.70±203.87	0.0015#

∧ Behavioral items and categories established by systematic ethogram of *M. fascicularis*. [14].

† Average duration in seconds per 30-minute observational phase.

* Mann–Whitney U test of means±S.D.’s. (*p*<0.05). To improve accuracy, the α-level was modified according to the quantity of variables (specifically, α′ = α/15 = 0.0033).

# *p≤*α′, demonstrating that the behavior was significantly modified by model.

Social plus visual isolation significantly reduced six behaviors in NVC subjects (in order of decreasing fold-change magnitude): ‘holding infant’ (a parental behavior), ‘lip smacking’ (a communication behavior), ‘presenting buttocks’ (a sexual behavior), ‘walking on shelf’ (a locomotive behavior), ‘nursing infant’ (a parental behavior), and ‘licking hair’ (a self-directed behavior) (Table 4). In contrast, social plus visual isolation significantly increased five behaviors (in order of decreasing fold-change magnitude): ‘hanging on window or door’ (a resting behavior), ‘licking residue from floor’ (an ingestion behavior), ‘grooming’ (an amicable behavior), ‘playing’ (a self-directed behavior), and ‘watching company’ (a vigilance behavior) (Table 4).

**Table 4:** Differential Behaviors in NVC Subjects Ranked by Fold-Change Magnitude.

Behavioral Item∧	Behavioral Category∧	Pre-isolation (s)†	Post-isolation (s)†	*P*-value*
Holding infant	Parental	146.38±396.02	48.28±213.80	0.0004#
Lip smacking	Communication	4.23±48.47	1.54±11.04	0.0000#
Presenting buttocks	Rutting and estrous (sexual)	3.32±18.90	1.51±8.76	0.0000#
Same-sex mounting	Rutting and estrous (sexual)	0.08±0.91	0.04±0.52	0.0253
Walking on shelf	Locomotive	5.29±12.64	3.15±7.81	0.0008#
Threatening	Conflict	0.48±4.12	0.30±3.99	0.0053
Nursing infant	Parental	12.16±56.98	9.10±53.46	0.0005#
Copulating	Mating (sexual)	0.64±2.96	0.49±2.06	0.0313
Licking hair	Self-directed	0.40±3.82	0.38±3.22	0.0006#
Licking tail	Self-directed	0.17±1.33	0.44±3.63	0.0460
Hanging on window or door	Resting	71.77±236.72	99.66±301.44	0.0004#
Licking residue from floor	Ingestion	3.47±30.73	4.32±33.60	0.0010#
Grooming	Amicable (affiliative)	85.88±185.11	94.13±205.99	0.0014#
Playing (solitary)	Self-directed	2.48±19.47	2.58±12.02	0.0000#
Watching company	Vigilance	1233.34±423.76	1258.14±424.27	0.0001#

∧ Behavioral items and categories established by systematic ethogram of *M. fascicularis*. [14].

† Average duration in seconds per 30-minute observational phase.

* Mann–Whitney U test of means±S.D.’s. (*p*<0.05). To improve accuracy, the α-level was modified according to the quantity of variables (specifically, α′ = α/15 = 0.0033).

# *p≤*α′, demonstrating that the behavior was significantly modified by model.

### Behavioral Effects of Social and Social Plus Visual Isolation Relative to Controls

To better assess the effects of social and social plus visual isolation, pre-isolation and post-isolation behaviors of SSC and NVC subjects were compared with those of non-isolated controls. As expected, there were no statistically significant differences between the pre-isolation subjects and controls (*p≥*0.05, Table 5).

**Table 5:** Differential Behaviors in SSC and NVC Subjects Versus Controls.

Behavior∧	Behavioral Category∧	CON (pre)†	Pre-Isolation†		*P-*value	CON (post)†	Post-Isolation†		*P-*value
			SSC	NVC			SSC	NVC	
Presenting buttocks	Rutting and estrous (sexual)	3.21±15.97	6.90±36.67	3.56±12.35	0.2034	1.75±9.74	0.00±0.00	0.00±0.00	0.03
Shaking ID card	Self-directed	0.44±5.33	0.09±0.61	0.26±1.90	0.7342	0.33±1.87	0.00±0.00	0.00±0.00	0.0292
Lying on floor	Resting	9.65±46.09	16.47±81.85	2.51±24.20	0.1120	72.00±199.26	54.25±193.29	16.49±77.33	0.0136
Same-sex mounting	Rutting and estrous (sexual)	0.08±1.01	0.00±0.00	0.00±0.00	0.5047	0.001±0.03	0.00±0.00	0.24±1.29	0.0002^#^
Being groomed	Amicable (affiliative)	100.75±194.74	79.42±153.26	68.20±149.51	0.1541	99.30±201.99	161.99±269.29	73.97±179.38	0.0059^#^
Embracing (with conspecific)	Amicable (affiliative), thermo-regulatory	279.19±463.93	278.95±405.63	271.43±415.23	0.9855	21.16±119.20	110.72±256.56	323.53±540.88	0.0000^#^
Huddling (solitary)	Self-directed,thermo-regulatory	346.13±423.97	296.35±334.62	358.58±375.34	0.4185	185.72±245.86	283.53±314.86	400.19±502.73	0.0000^#^

∧ Behavioral items and categories established by systematic ethogram of *M. fascicularis*. [14].

† Average duration in seconds per 30-minute phase. CON: non-isolated control group. As the pre-isolation and post-isolation groups were assessed during different seasons, it is only proper to compare pre-isolation data against the CON (pre) data and post-isolation data against the CON (post) data to control for seasonal variation. This phenomenon explains the variations between control (pre) and control (post) data. Pre-isolation: mixed analysis of variance (ANOVA), *p>*0.05; post-isolation: mixed ANOVA, *p<*0.05.

**Figure 1:**
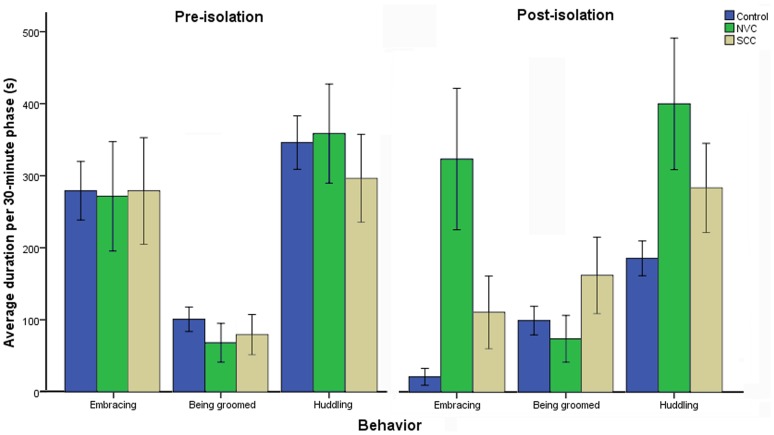
The significantly differentiated behaviors in SSC and NVC subjects versus controls. ‘Embracing’, ‘being groomed’, ‘huddling’, and ‘grooming’ were significantly differentiated between SSC, NVC, and controls by ANOVA (*p*≤α′). ‘Grooming’ was too insignificant in magnitude to be graphed here. To improve accuracy, the α-level was modified according to the quantity of variables (specifically, α′ = α/15 = 0.0033). See Table 5 for numerical data.

There were four behavioral items that were significantly differentiated in SSC and NVC subjects relative to controls (Table 5). ‘Same-sex mounting’ (a sexual behavior) was significantly reduced by social isolation relative to controls, but significantly increased by social plus visual isolation relative to controls. ‘Being groomed’ (an amicable behavior) was significantly increased by social isolation relative to controls, but significantly decreased by social plus visual isolation relative to controls. ‘Embracing’ (embracing a conspecific; an amicable and thermo-regulatory behavior) and ‘huddling’ (sitting alone on the floor with head down, displaying no interest in the external environment; a self directed behavior) was significantly increased by both social and social plus visual isolation relative to controls (Table 5, Fig. 1).

## Discussion

In this study, a depression-like behavioral phenotype, which may serve as a non-human primate model of depression, was constructed through 90-day social and social plus visual isolation randomly sampled from socially stable colonies. Differential behaviors from social and social plus visual isolation were identified by comparing mean durations of pre-isolation and post-isolation behaviors in SSC and NVC subjects and by comparing SSC and NVC subjects with non-isolated controls.

### Behavioral Effects of Social (SSC) and Social Plus Visual (NVC) Isolation

Social isolation significantly reduced one conflict behavior, ‘threatening’ (Table 3). ‘Threatening’ behavior is a dyadic antagonistic social behavior that has been well-established as an indicator of higher social status in adult female *M. fascicularis* [19] Michopoulos *et al.* have demonstrated that subordinate adult female *M. fascicularis* subjects receive more aggression and show more submission. [20] Shively *et al.* have demonstrated that social subordination is stressful, and depression-like behavior is more common in socially subordinate adult female *M. fascicularis* subjects. [21] Accordingly, antidepressant therapy has been shown to promote dominance in the dyadic social interaction paradigm in non-human primates and humans. [22].

Social and social plus visual isolation significantly reduced one communicative behavior, ‘lip smacking,’ a well-established core gesture in face-to-face interactions among macaques. [23] However, there are mixed interpretations as to its psychosocial meaning. One interpretation is ‘lip smacking’ signals submission or fearfulness, [24], [25] while another interpretation states that ‘lip smacking’ must accompany presentation of the buttocks to indicate submission; [26] in the least, as ‘lip smacking’ precedes affiliative interactions and is associated with grooming in almost every macaque species under investigation, it can been deemed a non-aggressive communicative behavior. [27], [28] The reduction in ‘lip smacking’ by social and social plus visual isolation indicates impairment in facial communication, a sign previously observed in depressed patients [29].

Social isolation significantly reduced one amicable (affiliative) behavior, ‘embracing’ (embracing a conspecific). Ventro-ventral embracing is an affiliative and bonding behavioral pattern commonly observed between macaque females living in captivity; [30] accordingly, antidepressant therapy has been shown to promote affiliative behavior in non-human primates and humans. [22] However, social isolation significantly increased ‘being groomed’ behavior, and social plus visual isolation significantly increased ‘grooming’ behavior. Grooming behavior has been extensively investigated as a socially affiliative behavior in macaques; interestingly, ‘grooming,’ as opposed to ‘being groomed,’ has been correlated with lower long-term stress levels in adult female macaques (or to borrow Shutt *et al.*’s language, “tis better to give than to receive.”). [31], [32] Thus, social plus visual isolation may produce lower long-term stress levels than mere social isolation. In sum, social isolation and social plus visual isolation appear to have differing effects on affiliative behavior.

Both social and social plus visual isolation significantly reduced one sexual behavior, ‘presenting buttocks’ (Tables 3, 4). Michopoulos *et al.* have also demonstrated that subordinate adult female *M. fascicularis* subjects receive less affiliation from other macaque subjects and exhibit decreased sensitivity to sexual hormonal stimulation (e.g., lower serum LH in response to estradiol and lower serum oxytocin). [20] Reding *et al.* also demonstrated that stress-induced attenuation of estradiol in adult female *M. fascicularis* subjects reduces sexual behavior and affiliation with male subjects. [33] Accordingly, sexual dysfunction is a common symptom of depression. [34] Thus, social and social plus visual isolation appears to diminish sexual interest, and social plus visual isolation appears to diminish sexual intercourse activity. Further investigation should focus on the comparative effects of social and social plus visual isolation on sexual behavior and hormones (e.g., estradiol, LH, oxytocin) in adult female macaques.

Social plus visual isolation significantly reduced two parental behaviors, ‘holding infant’ and ‘nursing infant’ (Tables 3, 4). These behavioral changes are consistent with those found in a previous 14-week *M. fascicularis* postpartum study, in which mother-infant contact, maternal holding, and infant suckling were all found to be decreased in individually-caged mothers relative to those in social groups. [35] As ‘holding infant’ and ‘nursing infant’ behaviors are indicators of mother-infant attachment in primates? [36] these findings suggest that social plus visual isolation reduces mother-infant attachment, which is similar to previous findings in depressed human mothers. [37] It should be mentioned that although none of the SSC or NVC subjects possessed their own offspring during this study on account of ethical restrictions, macaque females with no offspring do exhibit parental behaviors in the form of alloparental care (care of non-offspring). [38].

Social and social plus visual isolation significantly increased one self-directed behavior, solitary ‘playing’ (Tables 3, 4) and decreased one self-directed behavior, ‘licking hair.’ As to solitary ‘playing,’ non-social play is a well-established by-product of social isolation in human children and has been shown to be a risk factor for depression. [39], [40] As to decreased ‘licking hair’ behavior, self-grooming behavior has been shown to be adversely influenced in both rodent models of depression and depressed humans. [41], [42].

Social and social plus visual isolation significantly reduced one locomotive behavior, ‘walking on the shelf’ behavior (Tables 3, 4). Social plus visual isolation significantly increased one resting behavior, ‘hanging on window or door.’ Consistent with previous observations of the rhesus macaque, [43] our observations of the cynomolgus macaque in the free enclosure environment suggest that dominant individuals tend to reside on the ‘shelf’ (an elevated perch), and subordinate individuals tend to reside on the floor and/or the periphery of the free enclosure (window or door). This phenomenon may be a contributing factor to this finding.

Finally, social plus visual isolation significantly increased one ingestion behavior, ‘licking residue from floor,’ and significantly increased one vigilance behavior, ‘watching company.’ With respect to increased ‘licking residue from floor’ behavior, a rodent peer separation model of depression has also demonstrated abnormalities in eating behavior in isolated rats; [44] moreover, in humans, several eating disorder inventory (EDI) subscales have been shown to significantly correlate with depression severity with significant EDI decreases following antidepressant therapy. [45] With regard to increased ‘watching company’ behavior, previous work on depressed patients has also shown abnormalities in attentiveness; depressed patients have been found to exhibit selective attention to negative stimuli or appear to have “lost” the positive attentional bias that characterizes non-depressed individuals. [46].

### Behavioral Effects of Social and Social Plus Visual Isolation Relative to Controls

There were four behaviors that were significantly differentiated in SSC and NVC subjects relative to non-isolated controls: ‘same-sex mounting’, ‘being groomed’, ‘embracing,’ and ‘huddling’ (Table 5). ‘Same-sex mounting’, ‘being groomed,’ and ‘embracing,’ have been previously discussed. As to ‘huddling,’ previous studies have defined huddling behavior (either individually or in a group) as a thermo-regulatory behavior to conserve heat, [14], [47], [48] but solitary ‘huddling’ has also been interpreted as a depression-like posture in macaques. [11], [49] This behavior resembles the increased head flexion and thoracic kyphosis observed in MDD patients. [50].

There were two discrepancies between our significant pre- and post-isolation findings in the SSC and NVC groups (Tables 3, 4) and the significant differential findings in the SSC and NVC groups relative to non-isolated controls (Table 5). Contrary to the findings in pre- vs. post-isolation SSC subjects, ‘embracing’ was significantly increased by social isolation relative to controls. Contrary to the findings in pre- vs. post-isolation NVC subjects, ‘same-sex mounting’ was significantly increased by social plus visual isolation relative to controls. Both the pre-isolation and non-isolated environments were identical with respect to physical environment but differed with respect to subject consitution; specifically, the pre-isolation environment was composed of 30 subjects and the non-isolated control environment was composed of 20 subjects. This difference may have differentially influenced subject behavior.

### Social and Social Plus Visual Isolation of Macaques as a Model for Depression

MDD is a mood disorder that commonly presents in psychologically mature human adults capable of expressing a full range of human behaviors; therefore, an appropriate macaque model for depression should also be psychologically mature and without behavioral deficits. As previous macaque studies have established distinct developmental phases critical to behavioral acquisition, social or social plus visual isolation during these early-life developmental phases would produce life-long behavioral deficits that would be disadvantageous to constructing an appropriate non-human primate model for depression. Therefore, in this study, only psychologically mature adult female *M. fascicularis* subjects were selected for social and social,plus visual isolation.

In addition to developmental factors, the social environment is also critical in establishing a suitable macaque model of depression. In a single-cage solitary environment, behavioral expression is limited to simple, self-oriented, and stereotypic behaviors (e.g., self-clasping, self-grooming). However, the behaviors expressed in a small group environment of four to six interacting members are more diverse and social (e.g. playing, clinging, and mutual grooming with other subjects). Therefore, this social environment has become more popular in primate behavioral research; however, due to limited group membership, this environment still does not mimic natural social conditions. For example, the failure of Harlow’s monkeys to display relevant social behaviors may be attributable to the lack of social skill acquisition from a restricted social environment. [11] In this study, the macaque subjects were reared in a semi-natural group environment containing 18–24 members. In this larger social setting, macaques can express a broader range of social behaviors and better hone their social skills. [20] Thus, the adult subjects employed in this study were able to form a full complement of social skills and stable social bonds with other colony members prior to isolation. Although the behavioral patterns of socially and socially plus visually isolated subjects observed here resemble those found in Harlow’s work, [11] this study more conclusively illustrates that social and social plus visual isolation induces depression-like behavior by employing psychologically mature adult subjects reared in socially stable colonies. This experimental design precludes the lack of social skill acquisition or the absence of pre-existing social bonds as confounding factors.

## Conclusions

This study demonstrates that 90-day social and social plus visual isolation are effective in inducing depression-like behavior in psychologically mature adult female *M. fascicularis* subjects. This method can be applied to construct a non-human primate model of depression in order to assess physiological, behavioral, and social phenomena in a controlled laboratory setting. Through applying modern molecular techniques to this primate model of depression, the complex relationships between individual behavior, social dynamics, and the molecular etiopathology underlying depression can be investigated.

## Supporting Information

File S1 **Raw observational data in spreadsheet format.**
(XLS)
